# Impacts of arbuscular mycorrhizal fungi on nutrient uptake, N_2_ fixation, N transfer, and growth in a wheat/faba bean intercropping system

**DOI:** 10.1371/journal.pone.0213672

**Published:** 2019-03-11

**Authors:** Rosolino Ingraffia, Gaetano Amato, Alfonso Salvatore Frenda, Dario Giambalvo

**Affiliations:** 1 Department of Agricultural, Food and Forest Sciences (SAAF), University of Palermo, Palermo, Italy; 2 Angelo and Salvatore Lima Mancuso Foundation, University of Palermo, Palermo, Italy; Estacion Experimental del Zaidin, SPAIN

## Abstract

Arbuscular mycorrhizal fungi (AMF) can play a key role in natural and agricultural ecosystems affecting plant nutrition, soil biological activity and modifying the availability of nutrients by plants. This research aimed at expanding the knowledge of the role played by AMF in the uptake of macro- and micronutrients and N transfer (using a ^15^N stem-labelling method) in a faba bean/wheat intercropping system. It also investigates the role of AMF in biological N fixation (using the natural isotopic abundance method) in faba bean grown in pure stand and in mixture. Finally, it examines the role of AMF in driving competition and facilitation between faba bean and wheat. Durum wheat and faba bean were grown in pots (five pots per treatment) as sole crops or in mixture in the presence or absence of AMF. Root colonisation by AMF was greater in faba bean than in wheat and increased when species were mixed compared to pure stand (particularly for faba bean). Mycorrhizal symbiosis positively influenced root biomass, specific root length, and root density and increased the uptake of P, Fe, and Zn in wheat (both in pure stand and in mixture) but not in faba bean. Furthermore, AMF symbiosis increased the percentage of N derived from the atmosphere in the total N biomass of faba bean grown in mixture (+20%) but not in pure stand. Nitrogen transfer from faba bean to wheat was low (2.5–3.0 mg pot^-1^); inoculation with AMF increased N transfer by 20%. Overall, in terms of above- and belowground growth and uptake of nutrients, mycorrhization favoured the stronger competitor in the mixture (wheat) without negatively affecting the companion species (faba bean). Results of this study confirm the role of AMF in driving biological interactions among neighbouring plants.

## Introduction

Most terrestrial plants are able to establish symbiotic relationships with arbuscular mycorrhizal fungi (AMF) [1]. Such symbiosis plays an important role in: plant growth and yield; acquisition of nutrients [2], particularly P and other poorly mobile nutrients [3]; improved resistance to biotic stresses [4] and abiotic stresses (drought, salinity, etc.) [5]; and increased soil aggregation and carbon sequestration [6]. Furthermore, AMF can stimulate the growth of other microorganisms in the rhizosphere and alter the structure of the microbial community [7–9], influencing several biological process, including biological N fixation [10]. Arbuscular mycorrhizal symbiosis can improve root nodulation and N_2_ fixation by increasing the uptake of key nutrients (e.g., phosphorous) or by influencing legume–Rhizobium symbiosis [11,12]. The extent of these advantages, however, depends on the edaphic and climatic context in which symbiosis occurs, the AMF and plant species involved, and agricultural management practices (fertilisation, tillage, etc.). The influence of these factors is so great that under certain conditions the symbiotic relationship can turn into a parasitic one [13].

In intercropping systems, AMF create a hyphal network to connect root systems of neighbouring plants in the field, favouring the exchange of nutrients and carbohydrates among plants. One of the advantages of mixing legumes and non-legume species lies in the possibility of N transfer from the legumes to the companion crop. This transfer has been highlighted by several researchers, although it can vary widely, contributing by up to 80% of the N demand of the receiver plant [14]. In mixtures of legumes and non-legumes, an increase in N in the rhizosphere can occur through the decomposition of the thin roots and root nodules of the legume as well as through the release of radical exudates rich in soluble nitrogenous compounds from the legume [14]. A key role in N increase in the rhizosphere is played by the microbial community whose entity and structure is strongly influenced by the presence and composition of soil organic matter, e.g. thin roots and root nodules and root exudates [15]. The non-legume plants can intercept this N. Furthermore, mycorrhizal symbiosis can influence this process by intercepting, with high efficiency, the N made available and transferring it to the non-legume host plant for carbohydrates through the hyphal network, which connects the roots of nearby plants. The relative importance of each mechanism involved varies greatly depending on the characteristics of the agroecosystem, which may explain the variability in estimates of N transfer reported in the literature. In addition, discrepancies in results may be due to the different methods used to estimate N transfer. At any rate, the extent of the contribution of AMF to N transfer in mixtures of legumes and non-legumes is still unclear.

In the light of the above, we conducted the present research on durum wheat and faba bean grown in mixture or in pure stand and in presence or absence of AMF to address the following main questions: i) does the presence of AMF differently affect the nutrients uptake in the two species grown in mixture or in pure stand? ii) does the presence of AMF differently affect the N fixation process when the legume is grown in mixture or in pure stand? iii) do AMF affect the N transfer from the legume to the companion crop? and iv) which is the role of AMF in driving competition and facilitation between faba bean and wheat.

## Material and methods

### Experimental setup

Durum wheat (*Triticum durum* Desf., cv. Anco Marzio) and faba bean (*Vicia faba* L., cv. Dorenza) were grown in pots as sole crops or in mixture in the absence or presence of AMF (–myc and +myc, respectively). The experiment was conducted in a wire house protected from rain at the Pietranera farm (Agrigento province, Italy; the farm is property of the A. & S. Lima Mancuso Foundation–University of Palermo; no specific permissions were required for the trial location and the wire house study did not involve endangered or protected species) and followed a completely randomised design with five replicates. Each plastic pot (diameter = 20 cm, height = 50 cm) was filled with 14 kg of an artificial substrate composed by 30% in volume of agricultural perlite (Perlite Italiana, 1–2 mm in diameter) and by 70% in volume of 2 mm sieved agricultural soil; the agricultural soil had the following characteristics: 486 g kg^-1^ sand, 247 g kg^-1^ silt, 267 g kg^-1^ clay, 10.8 g kg^-1^ organic matter, pH 8, 0.86 g kg^-1^ total N, 0.065 g kg^-1^ P_2_O_5_, 0.135 g kg^-1^ K_2_O, 1.9 dS m^-1^ saturated electrical conductivity (25°C), water holding capacity 290 g H_2_O kg^-1^ soil, wilting point 180 g H_2_O kg^-1^ soil. The natural soil microbial community was extracted through filtration of a soil suspension. Briefly, a solution of 350 g soil per litre of distilled water was stirred for 20 min at 140 rpm; after decantation, the suspension was filtered through an 11 μm filter mesh to discard the natural AMF community. The solution was stored at 4°C until sowing time (the day after). The substrate was heat-sterilised at 130°C for 72 h.

Sowing was done in early January. The sole crops were distributed homogenously as 24 seeds per pot for durum wheat and 4 seeds per pot for faba bean. Ten days after emergence (DAE) the number of plants was thinned to 12 for wheat and 2 for faba bean. For both species, the final densities were those usually adopted by farmers in the Mediterranean environment (400 plants m^-2^ for durum wheat and 60 plants m^-2^ for faba bean). In the intercropping systems, a substitutive method was used; thus, the final number of plants per pot (after thinning) was six for durum wheat and one for faba bean.

At sowing time, 320 ml of the natural soil microbial community solution, obtained as previously described, was added to each pot. Simultaneously, pots in the +myc treatment were inoculated with 6 g of an inoculum composed of a mix of eight AMF species (*Gigaspora margarita*, *Funneliformis mosseae*, *Rhizophagus irregulare*, *G*. *clarum*, *G*. *deserticola*, *G*. *monosporum*, *G*. *brasilianum*, *G*. *aggregatum*) equally present at a density of 220 spores g^-1^ per each species.

To investigate possible N transfer from the legume to the non-legume, we prepared another set of 10 pots of the mixture treatment (five–myc and five +myc). We enriched the ^15^N content in the faba bean biomass in these pots by injecting labelled ammonium nitrate (NH_4_NO_3_, 98% of ^15^N content) directly into the faba bean stem. This was done in three applications of 200 μl each in equal concentrations (115 mM) starting at the beginning of wheat stem elongation (55 DAE); the second and third injections were done 66 and 73 DAE, respectively. A total of 1.925 mg N was injected per pot.

From sowing until the end of the experiment, we checked the soil moisture twice a week using the gravimetric method. When the soil moisture fell below 70% of the water holding capacity, tap water was added to bring the soil moisture back to the water holding capacity.

### Harvest and analysis

At wheat anthesis (85 DAE), all biomass was harvested. The aboveground biomass was oven-dried at 72°C until a constant weight, and the dry weight was recorded. We then ground the dry biomass to a fine powder and used it to quantify (a) total N using the Dumas method (flash combustion with an automatic N analyser; DuMaster D-480, Büchi Labortechnik, Flawil, Switzerland) and (b) total P, K, Na, Mg, Ca, Mn, Fe, Ni, Cu, and Mo using the Varian Vista-MPX simultaneous inductively coupled plasma optical emission spectrometer with charge-coupled device detection. Also, we determined the ^15^N content using a Roboprep-CN and 20–20 isotope ratio mass spectrometer (Europa Scientific, Crewe, UK).

The belowground biomass was extracted through sieving and subsequent washing. A root subsample was immediately cleared with KOH 10%, stained with trypan blue 0.05% [16], and used to quantify the percentage of AMF infection using the gridline-intersect method [17]. The rest of the root biomass was oven-dried at 70°C until a constant weight, and the dry weight was recorded. We used a representative root subsample (2 g; approximately 25–30% of the total root biomass) to determine the root length using the grid intersect method [18]. The roots of the two species in the mixture were tightly intertwined; therefore, a representative subsample of about 3 g (approximately 30% of the total root biomass) was extracted, and the roots of the two species were carefully separated. Hence, the total root biomass of each species was determined based on the known weight of: the overall subsample, the two single species, and the total pot root biomass.

#### Calculation

The total root length was calculated based on the known weight of both the subsample and the total root biomass. Also, we calculated the specific root length by dividing the total root length by the total root dry weight, and we obtained the soil root density by dividing the total root length by the pot soil volume.

We used the nutrient content to calculate the respective nutrient uptake by multiplying the content of each nutrient by the aboveground biomass. We used the ^15^N content to determine the percentage of legume N derived from symbiotic N fixation (%Ndfa) using the natural isotopic abundance method as described by Unkovich et al. [19] (Eq 1), and the amount of legume N derived from atmospheric fixation (Ndfa) was calculated according to Eq 2:

%Ndfa=δNw15-δNfb15δNw15-B×1001 (1)

$$Ndfa=N_{fb}\times \frac{\%Ndfa}{100}$$ (2)

where δ^15^N_w_ is the increment in ^15^N in the reference crop compared to in the air, δ^15^N_fb_ is the increment in ^15^N in the legume crop compared to in the air, B is the δ^15^N of faba bean grown in an N-free medium, and N_fb_ is N uptake in faba bean. As suggested by Unkovich et al. [19], we used 0.5 for B, which is the average value for faba bean.

To quantify N transfer from faba bean to wheat through direct labelling, we used the approach reported by Chalk et al. [20]. Specifically, the portion of faba bean N transferred to wheat (P_fb(→w)_) was determined according to Ledgard et al. [21] (Eq 3):

$$P_{fb(\to w)}=\frac{N_{w}\times E_{w}}{\left(N_{fb}\times E_{fb}+N_{w}\times E_{w}\right)}$$ (3)

where N_w_ is the wheat N uptake and E_w_ and E_fb_ are the ^15^N enrichment (% atom excess) for wheat and faba bean, respectively. P_fb(→w)_ was used to calculate the amount of N transferred from faba bean to wheat (N_fb(→w)_; Eq 4):

$$N_{fb(\to w)}=N_{fb}\times P_{fb(\to w)}$$ (4)

The proportion of wheat N derived from faba bean (P_w(←fb)_) was calculated as follows (Eq 5):

$$P_{w(\leftarrow fb)}=\frac{N_{fb(\to w)}}{N_{w}}$$ (5)

We obtained the percentage of faba bean N transferred to wheat (%N_fb(→w)_) and the percentage of wheat N derived from faba bean (%N_w(←fb)_) using Eqs 6 and 7, respectively:

$${\%N}_{fb(\to w)}=P_{fb(\to w)}\times \frac{100}{1}$$ (6)

$${\%N}_{w(\leftarrow fb)}=P_{w(\leftarrow fb)}\times \frac{100}{1}$$ (7)

#### Statistical analysis

Statistical analyses were performed with SAS statistical package [22]. We tested the effect of AMF inoculation (+myc, −myc) and cropping system (faba bean, wheat, mixture of faba bean and wheat) on the following parameters: above- and belowground biomass, leaf area, specific root length, and root density. For all other parameters, two-way analysis of variance was performed separately for the two species. All variables corresponding to proportions were arcsine-transformed before analysis to ensure a better fit with the Gaussian law distribution.

## Results

**Fig 1:**
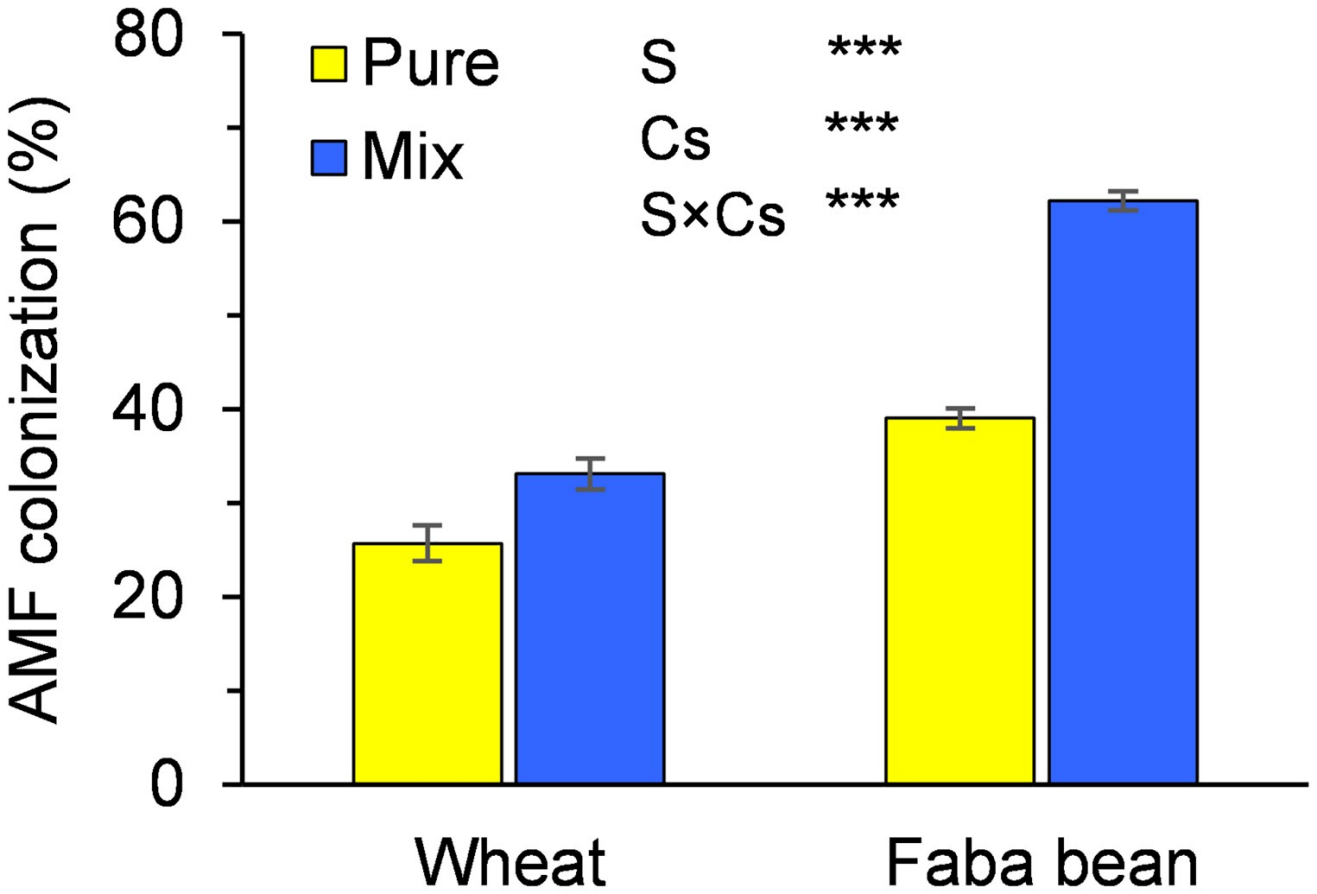
Root colonisation (%) by arbuscular mycorrhizal fungi in wheat and faba bean grown in pure stand (Pure) and in mixture (Mix). S, species; Cs, cropping system. *** indicates significant differences at P ≤ 0.001. Vertical bars indicate standard errors of each mean value (n = 5).

In the +myc treatment, greater colonisation was observed in faba bean than in wheat (51% and 29% on average, respectively; Fig 1). Intercropping positively influenced the root colonisation by AM fungi of both species (+47%); however, compared to the monocrops, the increase observed in faba bean (+59%) was significantly greater than that in wheat (+29%). In the–myc treatment, root colonisation was always less than 5%.

On average, the above- and belowground biomass of the mixture were much greater than those of the faba bean monocrop and similar to those of the wheat monocrop (Table 1). In the mixture, the contribution of wheat to the total biomass was much greater than that of faba bean, representing more than 90% of the aboveground biomass and more than 70% of the belowground biomass (Table 1). Therefore, in the mixture, the aboveground biomass of faba bean was, on average, about 5 g plant^-1^ compared to 11–12 g plant^-1^ in the pure stand; on the contrary, the biomass of wheat in mixture was approximately 10 g plant^-1^ vs 5 g plant^-1^ in the pure stand.

**Table 1:** Biomass, leaf area, and root traits in faba bean, wheat, and their mixture grown in the absence (–myc) or presence (+myc) of arbuscular mycorrhizal fungi. Cs, cropping system (faba bean, wheat, mixture); M, absence or presence of AMF.

		Faba bean		Wheat		Mixture		Cs	M	Cs × M
		-myc	+myc	-myc	+myc	-myc	+myc			
Above ground biomass	g pot^-1^	22.2	23.9	61.3	63.5	61.6 *(9)*^#^	63.7 *(7)*	***	†	ns
Below ground biomass	g pot^-1^	6.52	6.70	7.74	8.50	7.52 *(28)*	9.04 *(25)*	***	*	ns
Leaf area	cm^2^	1821	1858	1322	1342	1602 *(18)*	1627 *(17)*	**	ns	ns
Specific root length	m g^-1^	31.1	31.5	40.2	41.1	45.5	48.0	***	†	ns
Root density	cm cm^-3^	1.27	1.34	1.99	2.23	2.17 *(16)*	2.75 *(14)*	***	**	ns

^#^ In parenthesis percent contribution of faba bean on the total of the mixture. Data are means of 5 replicates.

^†^, *, ** and *** indicate significant differences at P ≤ 0.10, 0.05, 0.01, and 0.001, respectively; ns indicates differences not significant.

Compared to–myc, the AMF inoculum caused a modest increase in aboveground biomass production (+4% on average; P ≤ 0.10) but a larger increase in belowground biomass production (+11.3%; Table 1).

The total leaf area differed significantly by crop in the order faba bean > mixture > wheat, without appreciable differences due to mycorrhization. In all three crops, mycorrhization led to an increase in specific root length and, in particular, in root density (Table 1); in the mixture, the AMF inoculum significantly increased the root density of the two components (faba bean and wheat), although the effects were more pronounced in the cereal crop.

**Fig 2:**
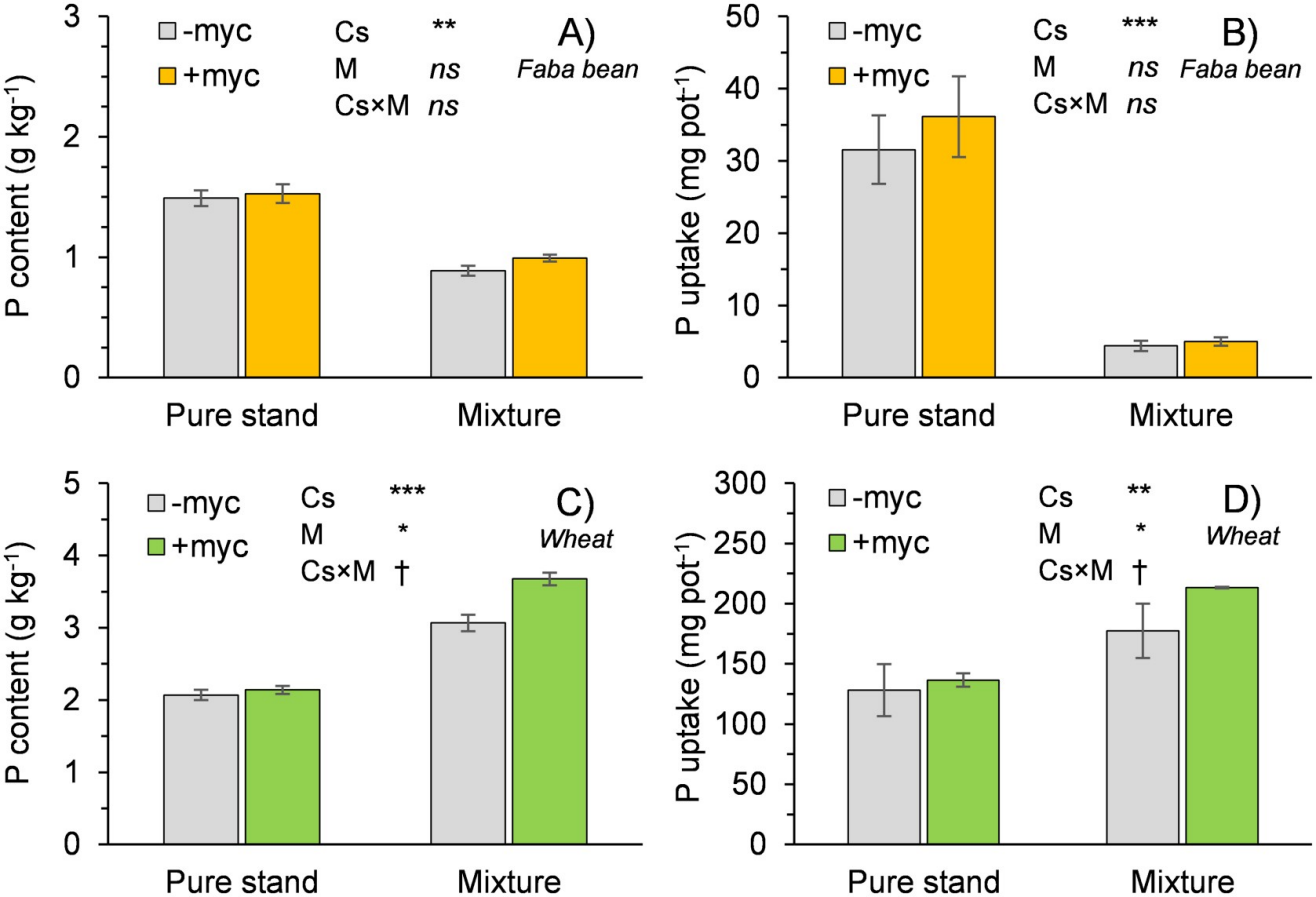
Phosphorus content and uptake of faba bean (A and B) and wheat (C and D) grown in pure stand and in mixture (values relative to aboveground biomass). Cs, cropping system (pure stand, mixture); M, absence (–myc) or presence (+myc) of arbuscular mycorrhizal fungi (AMF). †, *, **, and *** indicate significant differences at P ≤ 0.10, 0.05, 0.01, and 0.001, respectively; ns indicates nonsignificant differences. Vertical bars indicate standard errors of each mean value (n = 5).

The P content in the faba bean plants was significantly lower when the species was grown in mixture compared to pure stand, whereas the opposite was observed in wheat (Fig 2).

Considering both the relative P content and the biomass production obtained in pure stand and in mixture, the P uptake of faba bean grown in mixture was drastically reduced (–86%); in contrast, wheat plants grown in mixture showed a higher uptake compared to monocrops (+48%). AMF inoculation did not influence the faba bean plants either in monoculture or in mixture, whereas significantly increased P content and uptake were observed in wheat plants when grown in mixture.

**Fig 3:**
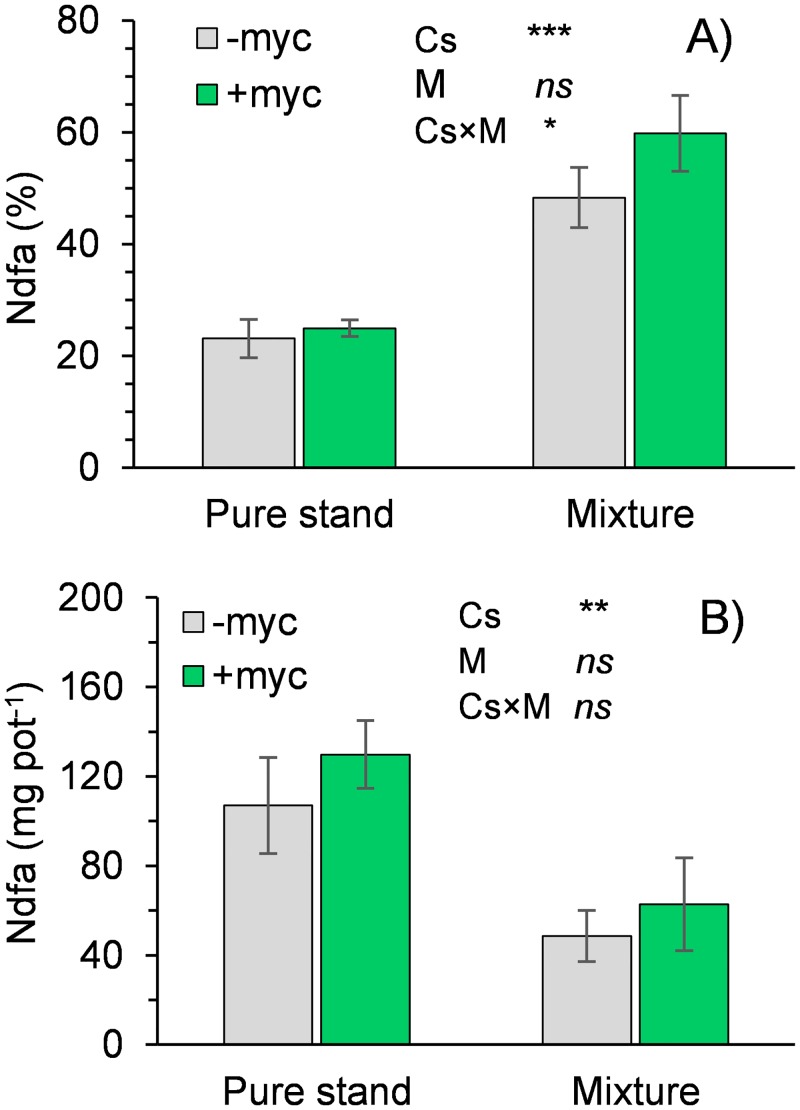
N derived from the atmosphere (Ndfa) as a percentage of total N content (A) and as the amount of N fixed (B) in the aboveground biomass of faba bean. Cs, cropping system (pure stand, mixture); M, absence (–myc) or presence (+myc) of arbuscular mycorrhizal fungi (AMF). *, **, and *** indicate significant differences at P ≤ 0.05, 0.01, and 0.001, respectively; ns indicates nonsignificant differences. Vertical bars indicate standard errors of each mean value (n = 5).

Faba bean grown in mixture had a lower N content than plants grown in pure stand (–10%; Table 2), whereas a slight increase was observed in mixed wheat compared to the wheat monocrop (+6%; Table 3). N content was not influenced by AMF inoculation (Tables 2 and 3). %Ndfa in faba bean increased greatly when plants were grown in mixture with wheat compared to as monocrops (Fig 3A); however, because of the very different amounts of biomass accumulated by faba bean plants in the two cropping systems, the total Ndfa was greater in the monoculture than in the mixture (Fig 3B). Mycorrhizal inoculation produced a small incremental change in N fixation (as percentage of total N content) when faba bean was intercropped but not when it was grown in pure stand.

**Table 2:** Contents of macro- and microelements in faba bean grown in pure stand or in mixture in the absence (–myc) or presence (+myc) of arbuscular mycorrhizal fungi. Cs, cropping system (pure stand, mixture); M, absence or presence of AMF.

		Pure stand		Mixture		Cs	M	Cs × M
		-myc	+myc	-myc	+myc			
N	g kg^-1^	20.5	21.6	19.2	19.0	**	ns	ns
K	g kg^-1^	20.0	20.7	26.6	25.6	***	ns	ns
Ca	g kg^-1^	35.8	33.6	29.6	31.4	ns	ns	ns
Mg	g kg^-1^	4.43	4.49	3.44	3.61	**	ns	ns
Na	g kg^-1^	2.79	3.11	2.75	2.46	ns	ns	ns
Fe	mg kg^-1^	328	296	259	262	ns	ns	ns
Mn	mg kg^-1^	97.4	93.8	76.9	96.9	ns	ns	ns
Zn	mg kg^-1^	39.1	37.8	30.4	30.3	*	ns	ns
Mo	mg kg^-1^	19.6	18.1	15.9	16.1	*	ns	ns
Ni	mg kg^-1^	2.57	4.14	2.34	3.01	ns	ns	ns
Cu	mg kg^-1^	6.64	7.63	6.21	6.54	ns	ns	ns

Data are means of 5 replicates.

*, ** and *** indicate significant differences at P ≤ 0.05, 0.01 and 0.001, respectively; ns indicates differences not significant.

**Table 3:** Contents of macro- and microelements in wheat grown in pure stand or in mixture in the absence (–myc) or presence (+myc) of arbuscular mycorrhizal fungi. Cs, cropping system (pure stand, mixture); M, absence or presence of AMF.

		Pure stand		Mixture		Cs	M	Cs × M
		-myc	+myc	-myc	+myc			
N	g kg^-1^	12.2	12.2	13.1	12.8	†	ns	ns
K	g kg^-1^	21.3	21.8	24.4	23.3	†	ns	ns
Ca	g kg^-1^	3.94	4.50	3.87	3.48	ns	ns	ns
Mg	g kg^-1^	0.82	0.86	0.85	0.79	ns	ns	ns
Na	g kg^-1^	0.74	0.30	0.87	0.66	ns	†	ns
Fe	mg kg^-1^	319	501	263	383	**	*	ns
Mn	mg kg^-1^	100.0	120.2	111.6	106.2	ns	ns	ns
Zn	mg kg^-1^	30.4	38.5	56.2	61.7	**	*	ns
Mo	mg kg^-1^	2.28	1.77	1.97	2.07	ns	ns	†
Ni	mg kg^-1^	2.96	3.62	2.77	2.45	ns	ns	ns
Cu	mg kg^-1^	4.04	3.17	10.06	7.55	*	ns	ns

Data are means of 5 replicates.

^†^, *, ** and *** indicate significant differences at P ≤ 0.10, 0.05, 0.01 and 0.001, respectively; ns indicates differences not significant.

**Fig 4:**
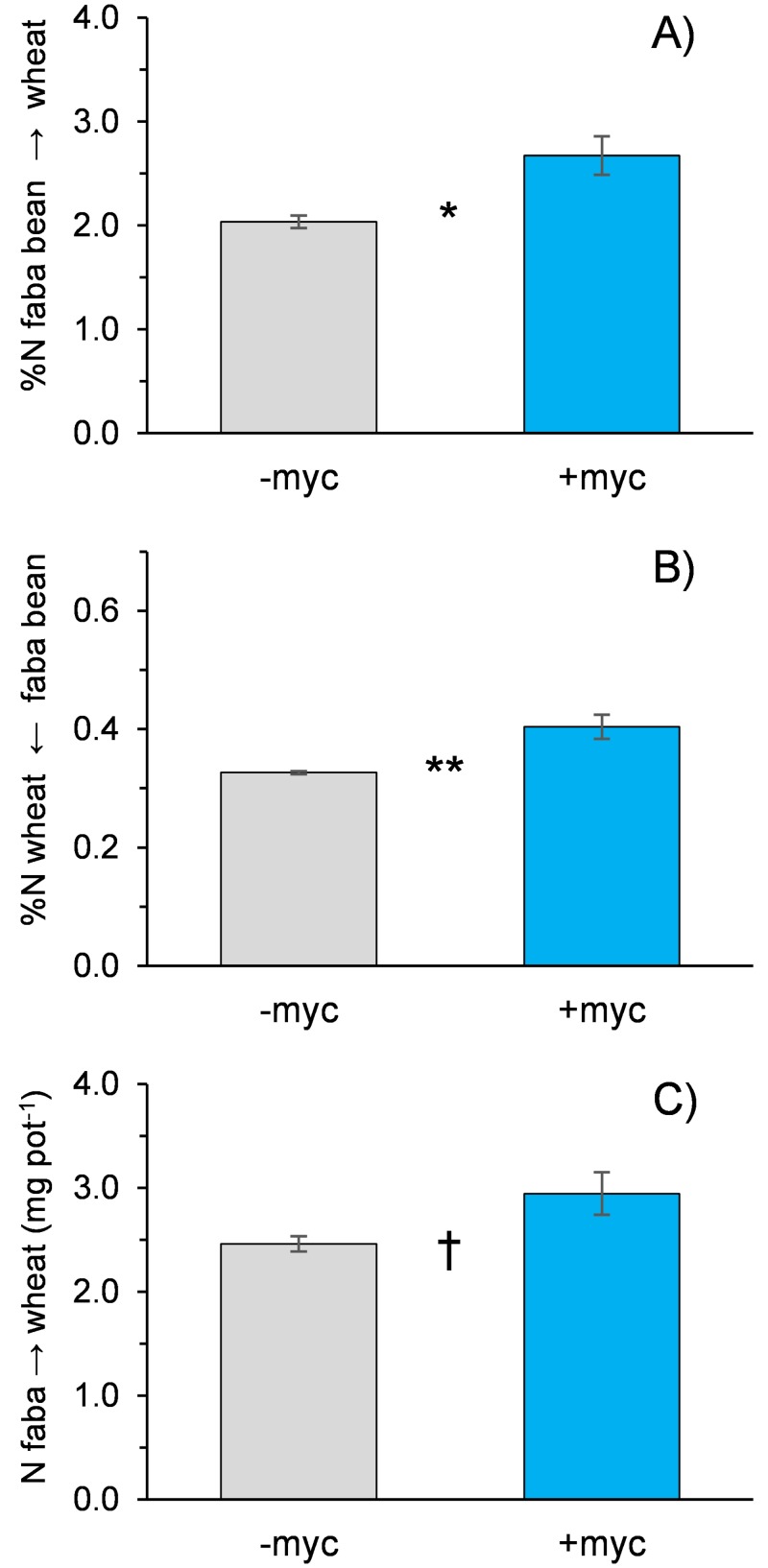
Percentage of N transferred from faba bean to wheat (A), percentage of wheat N derived from faba bean (B), and amount of N transferred from faba bean to wheat (C) in the absence (–myc) or presence (+myc) of arbuscular mycorrhizal fungi. †, *, and ** indicate significant differences at P ≤ 0.10, 0.05, and 0.01, respectively. Vertical bars indicate standard errors of each mean value (n = 5).

N transfer from faba bean to wheat was detected both with and without the AMF inoculum. The presence of AMF significantly increased the percentage of faba bean N transferred to the cereal (from 2.03% to 2.67%; Fig 4A) as well as the percentage of N in wheat directly derived from faba bean (from 0.33% to 0.40%; Fig 4B). The amount of N transferred from legume to non-legume was 2.46 and 2.94 mg pot^-1^ in–myc and +myc, respectively (P < 0.1; Fig 4C).

Intercropping increased the K content of the biomass compared to both monocultures; however, the responses of the two mixed species differed (+29% in faba bean and +11% in wheat on average; Tables 2 and 3). In contrast, intercropping reduced the Mg content of faba bean and had no effect on wheat plants.

Like the N content, the K, Ca, and Mg contents of both species and their mixture were not influenced by AMF colonisation (Tables 2 and 3). The inoculum affected the Fe and Zn content only in wheat, particularly when grown in pure stand. No effects were observed for either species for the other micronutrients studied (Mn, Mo, Ni, Cu; Tables 2 and 3).

## Discussion

Mycorrhizal colonisation was greater in faba bean than in wheat; this result is in line with the findings of Powell and Sithamparnathan [23] and Barea et al. [24] that legumes are in general more mycotrophic than grasses. Although AMF are usually non-host-specific, some species depend to a greater extent on mycorrhizal symbiosis for their growth than others [25]. In this experiment, greater arbuscular mycorrhizal colonisation was observed in the intercropped species than in the monocrops. This result agrees with the findings of other studies [24,26–27]. According to Smith and Read [1], many factors contribute to regulating AMF colonisation, such as root density, root exudates, and the availability of nutrients in the rhizosphere. In this study, an increase in root density was observed in the mixture compared to the monocrops (either wheat or faba bean); considering this it is also possible to hypothesize an increase in exudates as the two parameters are more or less closely associated as shown in some researches [28]. The root exudates govern signalling between AMF and host plants [29], stimulating the activation of AMF symbiosis. Such mechanism has also been highlighted by de Araujo Pereira et al. [30] who found a significant increment of root colonization in *Eucalyptus* grown in presence of *Acacia* plants suggesting a possible stimulation of the symbiosis in *Eucalyptus* root when in consortium. Moreover, the same authors have suggested that the presence of the legume in the mixture could stimulate the alkaline phosphatase activity in the soil resulting in an increase of the AMF dynamics. In addition, Rachid et al. [31] and de Araujo Pereira et al. [32] have reported that the presence of the legume can promote a greater diversity of AMF and soil bacterial communities by increasing the organic matter and the availability of N into the soil with a positive impact on the root colonization.

The increase in root colonisation in the intercropped plants was lower in wheat than in faba bean; the legume was the weaker competitor for nutrients and other resources, and its difficulties satisfying its own needs may have increased its dependence on symbiosis to overcome its own limits.

According to several researchers [2,3,33], mycorrhization favours root growth both in monocrops (+3% for faba bean root biomass and +10% for wheat) and in mixture (+20%); similar trends, but of much lower intensity, have been observed for aboveground biomass. In the mixture, wheat was the most competitive component, contributing on average more than 90% to the aboveground biomass and about 75% to the root biomass.

In this experiment, inoculation with AMF favoured (albeit slightly) wheat, which was the stronger component of the mixture, without negatively affecting the development of the legume. This result is unexpected, as several studies on natural plant communities have found that AMF reduce disparities in competitiveness between dominant and subordinate plant species [34–35]. Also, in contrast to what was found in the present experiment, Qiao et al. [2] showed that AMF mediated wheat/faba bean competition by acting in favour of the legume, which in their experiment was the weaker component. Other factors, such as soil characteristics, the availability of nutrients, differences in AMF and plant species, plant density ratios, and so on, might generate different effects of arbuscular mycorrhizal symbiosis on interspecific plant interaction and, as a consequence, the plant community [36]. The understanding of the mechanisms through which mycorrhizal symbiosis influences the interactions between associated plant components would be essential also in order to identify technical and management methods able to favourably guide, from an agronomic point of view, the interactions themselves.

AMF inoculation had different effects on the two species in relation to both the concentration and the uptake of P. In fact, mycorrhizal symbiosis resulted in a significant increase in the content and uptake of P in wheat, in line with what has been observed in many other studies [37], but only when the species was grown in mixture. The presence of the legume presumably increased the P available in the substrate, as highlighted by Hinsinger et al. [38], particularly around the legume roots, and AMF favoured its interception and subsequent transfer to the cereal, which, in the mixture, was the stronger component and therefore able to transfer carbohydrates to AMF.

Contrary to what has been observed in wheat, mycorrhizal symbiosis in faba bean did not have significant effects on P concentration and uptake. This result contrasts with what is reported in the literature for many legumes [33,39], that mycorrhizal symbiosis increases the uptake of P, which is notoriously one of the main factors limiting the development and production of this group of species. However, the advantage offered by AMF symbiosis increases with decreasing availability of P in the substrate [40,41]. Most likely, in this experiment, the availability of P was not limiting for the legume; this is confirmed by the fact that P removal of the faba bean monocrop was 3–4 times lower than that of the wheat monocrop; so faba bean, to meet its needs, used only a portion of P available in the substrate. In the faba bean grown in mixture, the content of P in the biomass and the overall uptake were much lower than in the monocrop, which further underlines the difficulties the species has in competing with the cereal component; also, in this case, mycorrhization did not appear to be able to offer an advantage to the legume. Moreover, it is possible that a certain amount of P could have been transferred, via AMF, from faba bean to wheat, a mechanism highlighted by Yao et al. [33].

Mycorrhizal symbiosis positively influenced the N fixation process in faba bean grown in mixture but not in pure stand. Given the characteristics of the substrate used in this experiment, it is likely that the amount of N available in the soil was sufficient to meet the needs of the faba bean grown in pure stand. This may also explain the significantly lower value for observed %Ndfa (24% on average) compared to those reported for the species in reviews by Jensen et al. [42] (range = 34–99%) and Ruisi et al. [43] in a typical Mediterranean environment (range = 50–93%). In contrast, in the mixture, faba bean was forced to increase rhizobial symbiosis to satisfy its needs for N, as the non-legume component depleted N from the soil. In such limiting conditions for the legume, mycorrhizal symbiosis seems to play a relevant role. After all, several studies have shown that the advantages of arbuscular mycorrhizal symbiosis in terms of plant growth and nutrient uptake are particularly evident when plants are grown under nutrient-limiting conditions [44–46].

Mycorrhization increased the contents of Fe and Zn in wheat, in line with previous research [47–49], but not those of faba bean. AMF alter in different ways the plant’s acquisition of Fe based on the soil characteristics and the effectiveness of the root system in terms of absorption [50]. According to Clark and Zeto [51], when levels of nutrients are low and in alkaline soil (like the soil in the present experiment), mycorrhizae hyphae enhance Fe uptake. The different behaviours of wheat and faba bean could be related to the different abilities of the plants to acidify the rhizosphere (an ability that is generally greater in legumes than cereals [52,53]) and make Fe available in subalkaline soils, as well as to the different needs of each species related to the different development of the root system (which is greater in wheat than in faba bean). It is likely that under the present experimental conditions, faba bean, by virtue of the characteristics of its root system, was able to meet independently its own Fe needs. In contrast, Fe could have been a limiting factor for wheat growth, and under these conditions mycorrhizal symbiosis could have enhanced Fe uptake and plant growth. Similar conclusions can be made regarding the concentration of Zn.

The strong increase in Fe and Zn contents observed in durum wheat biomass suggests, according to Ercoli et al. [3], that inoculation with mycorrhizal fungi may be of great interest in terms of the production of biofortified foods. This is even more significant because the diets of more than 60% and 30% of the world population are deficient in Fe and Zn, respectively [54].

To estimate N transfer, we used direct labelling (^15^N labelling); this very sensitive method is preferable to other methods because it permits yield-independent estimations. Results showed N transfer from faba bean to wheat even if its magnitude was lower than observed in other studies [55,56]. The short growing period and relatively short time from labelling to harvest may have contributed to the low values for N transfer. Furthermore, faba bean growth was greatly reduced by the competition with wheat, and this reduced the contribution of the faba bean to the fulfilment of the N demand of the companion wheat. Inoculation with AMF increased by 20% the amount of N transferred from faba bean to wheat. Li et al. [26] showed that N transfer from mung bean to rice increased from 5.4 to 15.7% because of hyphal linkage. In our experiment, we observed greater mycorrhization in the mixture than in the two pure stands; this cannot fail to have ecological implications and suggests that mycorrhizal symbiosis can offer great advantages in intercropping systems. A greater diversification of the plant biotic components also has advantages for AMF that can activate symbiotic relationships with multiple partners (each with its own specificities and able to provide diversified contributions for fungal growth), interconnect their root systems, and thus favour the exchange of nutrients among neighbouring plants. However, it is not possible to rule out the idea that mycorrhizae favoured the non-legume intercropped species by improving acquisition of N released by root exudates and the mineralisation of legume nodules and fine roots.

In addition, AMF could also have contributed to N transfer indirectly by stimulating soil bacteria involved in the mineralisation processes of soil organic matter, as observed by Saia et al. [46], and therefore also of plant tissues and nodules of the legume. Moreover, mycorrhizae can affect carbon flow to different components of the soil ecosystem able to direct the biological processes through the production of enzymes, organic acids, and other compounds, which can directly and indirectly promote the formation and stability of the aggregates [57] and the degradation of organic substrates or the solubilisation of mineral substrates [58], so indirectly favouring N transfer. A deeper understanding of the importance of each pathway involved in AMF-mediated N transfer is essential to accurately defining strategies to manage the soil/plant system to improve this important ecological process. This will require new and creative research approaches.

Overall, this experiment highlights the fact that arbuscular mycorrhizal symbiosis have an important role in the growth of wheat grown both in pure stand and mixed with faba bean, favouring the uptake of some macro- and micronutrients. Furthermore, mycorrhization enhances the percentage of N derived from atmosphere of faba bean grown in mixture and N transfer from the legume to the companion crop. Finally, mycorrhization in mixture favours the stronger competitor (wheat) without negative consequences for the weaker component (faba bean) and plays an ecological role by driving biological interactions among neighbouring plants. It is also interesting to recall how the faba bean in the mixture had a greater root AMF colonization than in the pure stand; this probably happened in response to the lack of resources for the legume, induced by the presence of the cereal proved to be much more able to use the available resources. However, the high mycorrhization level in the intercropped faba bean did not allow an advantage for the legume itself; probably the potential advantages offered by the increase in mycorrhization have been counterbalanced by the higher cost of the symbiotic relationship for the legume itself and/or by an increase in nutrient transfer mediated by fungal hyphae, which, as mentioned, has favoured the strongest component, able to support the mycelium with a carbohydrate flow.

The mechanisms through which AMF alter the competitive relationship between companion plants are still unclear. Knowledge of these mechanisms is essential for agronomically enhancing the role that AMF are able to perform.
